# Host Diversity and Potential Transmission Pathways of SARS-CoV-2 at the Human-Animal Interface

**DOI:** 10.3390/pathogens10020180

**Published:** 2021-02-08

**Authors:** Hayden D. Hedman, Eric Krawczyk, Yosra A. Helmy, Lixin Zhang, Csaba Varga

**Affiliations:** 1 Summit County Local Public Health Agency, Summit County, Frisco, CO 80443, USA; Hayden.Hedman@summitcountyco.gov; 2 Department of Microbiology and Immunology, University of Illinois Chicago, Chicago, IL 60612, USA; ekrawc4@uic.edu; 3 Food Animal Health Research Program, Department of Veterinary Preventive Medicine, Ohio Agricultural Research and Development Center, The Ohio State University, Wooster, OH 44691, USA; helmy.6@osu.edu; 4 Department of Epidemiology and Biostatistics, Michigan State University, East Lansing, MI 48824, USA; lxzhang@epi.msu.edu; 5 Department of Microbiology and Molecular Genetics, Michigan State University, East Lansing, MI 48824, USA; 6 Department of Pathobiology, College of Veterinary Medicine, University of Illinois Urbana-Champaign, Urbana, IL 61802, USA

**Keywords:** coronavirus, SARS-CoV-2, host diversity, One Health, COVID-19, animals, humans

## Abstract

Emerging infectious diseases present great risks to public health. The novel severe acute respiratory syndrome coronavirus 2 (SARS-CoV-2), causing coronavirus disease 2019 (COVID-19), has become an urgent public health issue of global concern. It is speculated that the virus first emerged through a zoonotic spillover. Basic research studies have suggested that bats are likely the ancestral reservoir host. Nonetheless, the evolutionary history and host susceptibility of SARS-CoV-2 remains unclear as a multitude of animals has been proposed as potential intermediate or dead-end hosts. SARS-CoV-2 has been isolated from domestic animals, both companion and livestock, as well as in captive wildlife that were in close contact with human COVID-19 cases. Currently, domestic mink is the only known animal that is susceptible to a natural infection, develop severe illness, and can also transmit SARS-CoV-2 to other minks and humans. To improve foundational knowledge of SARS-CoV-2, we are conducting a synthesis review of its host diversity and transmission pathways. To mitigate this COVID-19 pandemic, we strongly advocate for a systems-oriented scientific approach that comprehensively evaluates the transmission of SARS-CoV-2 at the human and animal interface.

## 1. Introduction

Coronaviruses (CoVs) (order: Nidovirales, family: Coronaviridae, subfamily: Coronavirinae) are enveloped, positive-stranded RNA viruses [1,2,3,4]. CoVs can infect birds (*Gammacoronaviruses* and *Deltacoronaviruses*) or mammals (predominantly *Alpacoronaviruses* and *Betacoronaviruses*) [5,6]. For over 80 years, animal coronaviruses, such as transmissible gastroenteritis virus (TGEV) of swine or bovine CoV (BCoV), have been known to infect wildlife and livestock species [7]. To date, seven CoVs have been identified in humans: HCoV-OC53, HCoV-229E, HCoV-NL63, HCoV-HKU1, MERS-CoV, SARS-CoV, and SARS-CoV-2. The first reports of endemic human CoVs (HCoVs) were documented in the 1960s when HCoV-OC53 and HCoV-229E were described [8,9]. It was not until 2004 and 2005 that HCoV-NL63 and HCoV-HKU1 were detected, respectively [10,11]. Endemic human coronaviruses most likely evolved from ancestral viruses of animal reservoirs [6,12].

In 2003, severe acute respiratory syndrome coronavirus (SARS-CoV) was reported as the first CoV of global health importance, which originated from several horseshoe bat species before transmission into human populations [13]. At least 8000 infections and 774 mortalities were linked to SARS-CoV [14]. Less than a decade later, the Middle Eastern respiratory syndrome (MERS) illness caused by a coronavirus (MERS-CoV) became an endemic disease throughout the Middle East, Africa, and Southeast Asia [15]. The zoonotic origins of MERS-CoV remain unclear, but it is speculated that the virus was transmitted from bat species to dromedary camels in the distant past [15,16].

In December 2019, severe acute respiratory syndrome coronavirus 2 (SARS-CoV-2) was first detected in the Huanan South seafood market (HSSM), a large market that also traded live animals, within Wuhan City, Hubei province, China [17]. In addition to fish and shellfish, a diverse selection of live wildlife, including hedgehogs, badgers, snakes, and poultry, was marketed at the time when the outbreak occurred [18]. Aside from live wildlife, animal-food products such as carcasses and meat were also available [19]. Here, it is suggested that several clusters of pneumonia cases were linked to the HSSM [20,21]. Phylodynamic analysis reveals that SARS-CoV-2 most likely emerged as early as October 2019 [22,23], suggesting that the HSSM was mainly a super spreading location and not an index spillover event. Shortly after, SARS-CoV-2 spread globally and the World Health Organization (WHO) declared it a pandemic on 11 of March 2020 [24]. As of 4 of January 2021, approximately 1,839,660 million people have died from the novel coronavirus disease 2019 (COVID-19) and more than 83,910,386 million have been infected worldwide [24]. Following 229E-CoV, NL63-CoV, OC43-CoV, HKU1-CoV, SARS-CoV, and MERS-CoV, SARS-CoV-2 is the seventh coronavirus to infect humans.

SARS-CoV and SARS-CoV-2 belong to the subgenus *Sarbecoviruses*, characterized by frequent recombination events [25,26]. To date, research indicates that SARS-CoV-2 is not an outcome of a recombination event of any known *Sarbecoviruses* [27]. It is hypothesized that SARS-CoV-2 originated from an unknown animal reservoir [20,21,28,29,30]. Currently, the closest related sequences originated from horseshoe bat (96%) and pangolin CoVs (91%) [31,32]. Although the receptor-binding domain (RBD) between pangolin CoV is structurally identical to SARS-CoV-2 [33,34], it is unclear if pangolins function as intermediate or dead-end hosts [35,36,37]. Moreover, a diverse array of mammalian, avian, and reptilian species have been proposed as other potential intermediate hosts [38,39,40,41] Narrow genomic variation in CoVs can lead to wide host diversity as demonstrated by the similarity of SARS-CoV-2 to SARS-CoV and MERS-CoV, sharing 99.8% [36] and 99.5% [42] similarity to that from civet cats and dromedary camels, respectively. Consequently, minimal genetic variation is needed for CoVs to exhibit unique host specificity. Therefore, numerous mammalian, avian, and reptilian species have been proposed as potential hosts of SARS-CoV-2 [38,39,41,43,44,45].

Here, we provide an overview of the host diversity SARS-CoV-2 to veterinary and public health interventions. Evidence in support of reverse zoonotic transmission has been reported in numerous settings where infected humans have engaged in close contact with domestic and captive zoo animals [40,46]. Mink is the only animal to date that has been shown to transmit SARS-CoV-2 to humans, however, we cannot exclude a SARS-CoV-2 transmission potential from cats, dogs, and ferrets to humans. Further studies are needed to elucidate this hypothesis. Moreover, in selected animal groups, there is evidence that animals were infected by SARS-CoV-2 from humans, followed by a subsequent zoonotic transmission of SARS-CoV-2 from these same animals back to human populations [46,47]. This review aims to provide a cross-disciplinary, “One Health” approach to evaluate the SARS-CoV-2 emergence and spread at the intersection of humans and animals [38]. Based on the definition from the Centers for Disease Control and Prevention (CDC): “One Health is a collaborative, multisectoral, and transdisciplinary approach—working at the local, regional, national, and global levels—with the goal of achieving optimal health outcomes recognizing the interconnection between people, animals, plants, and their shared environment” [48]. Furthermore, these findings might support future surveillance programs to unravel the complex evolutionary histories of SARS-CoV-2 and those of SARS-CoV-like CoV viruses of other animal host species.

## 2. Epidemiology of Human SARS-CoV-2 Infections

Contextual understanding of the epidemiology of the virus is essential to properly study the epidemiology of SARS-CoV-2. Since the initial outbreak in Wuhan, most research on SARS-CoV-2 transmission has been collected through human-to-human transmission studies [49]. Initial studies of SARS-CoV-2 have indicated that the reproductive number (R_0_) in humans varies from 1.4 to 3.9 [50,51,52,53,54], and approximately 40 to 50% of SARS-CoV-2 human cases are asymptomatic [55,56,57,58,59]. The incubation period for COVID-19 is speculated to be 14 days alongside a median time of 4–5 days from exposure to symptoms onset [60,61,62]. Global disease trends suggest that women exhibit stronger immune responses than men and they have lower mortality rates [63,64]. Moreover, living at high altitudes has been suggested as a potential natural protective effect for lower mortality [65,66]. Additionally, viral transmission varies by geographic region due to differences in cases’ demographics, genetics, and health behavior practices [36,53,67].

At the population level, systematic health and socioeconomic inequalities have placed many marginalized groups at increased risk of high morbidity and mortality of SARS-CoV-2 infections [68,69]. Previous studies documented that racial and ethnic minorities are disproportionately higher affected by SARS-CoV-2 infections [70,71]. In many of these cases, social determinants have historically limited these groups from accessing fair opportunities for economic, physical, and emotional health [72]. Moreover, socioeconomic status has been linked to the availability of housing and housing conditions (i.e., the number of individuals per household) [73,74]. Living conditions, such as homelessness and crowded living environments (e.g., prisons, nursing homes, and orphanages) have been reported to be associated with increased SARS-CoV-2 infections [75,76].

At the individual level, older adults and people with underlying medical conditions are at higher risk for a severe SARS-CoV-2 illness [77]. In contrast to these groups, most infected children that express symptoms, if any, are generally mild and require only supportive care [56,78]. According to the CDC, some examples of underlying medical and physical conditions that could increase the risk of severe SARS-CoV-2 illness include cancer, chronic kidney disease, heart conditions, obesity (body mass index (BMI) of 30 kg/m^2^ or higher but <40 kg/m^2^), severe obesity (BMI ≥ 40 kg/m^2^), and diabetes mellitus [77]. Other individual-level risk factors include people with disabilities, developmental and behavioral disorders, and drug and substance use disorders [77].

## 3. Transmission Pathways of SARS-CoV-2 in Humans

Transmission of SARS-CoV-2 among humans is thought to occur via three primary pathways: (1) inhaling respiratory droplets from an infected individual, (2) inhaling infected airborne particles, (3) or contact with infected environmental surfaces also known as fomites [79]. Indirect or direct contact with infected people can facilitate the exposure to infected saliva and other respiratory secretions, commonly excreted when an infected individual coughs, sneezes, talks, or sings [80,81,82,83]. It is important to note that the diameter of respiratory droplets (>5–10 μm) is typically larger than that of nuclei or aerosols (>5 μm) [84]. Therefore, the transmission of infected respiratory droplets can occur when a susceptible individual is within 1 m of an infected case [85].

In contrast to droplet transmission, airborne transmission can occur mostly indoors through the dissemination of infectious aerosols that can be suspended in the air for long distances (usually greater than 2 m) and periods (typically hours) [86,87]. Experimental studies that have created infectious aerosols in controlled laboratory settings demonstrated that SARS-CoV-2 can persist in the air from 3 to 16 h [87,88]. Additionally, respiratory excretions from infected individuals can contaminate a variety of surfaces, thus creating fomites that can infect other individuals in the immediate environment upon contact followed by touching the mouth, nose, or eyes [85].

In general, microenvironmental characteristics such as ambient temperature, pH, and humidity greatly impact the persistence of SARS-CoV-2 on surfaces [89]. Similar to other human and animal CoVs [89], SARS-CoV-2 also exhibits low persistence on copper, latex, and other limited porosity surfaces compared to metals, glass, and highly porous fabrics [90,91]. Although SARS-CoV-2 has been reported to survive in environments at 40 °C for up to several hours [92], CoVs survive best at lower environmental temperatures and lower relative humidity [89]. While at room temperature, SARS-CoV-2 is stable at a wide range of pH values (pH 3–10) [93]. Despite evidence of SARS-CoV-2 contamination of surfaces and persistence on various substrates, there is no specific study that directly associates SARS-CoV-2 transmission through fomites [85]. Therefore, it is suggested that fomite transmission has lower importance compared to transmission via inhaling infected respiratory droplets or airborne particles [93,94].

Other modes of SARS-CoV-2 transmission could potentially include fecal-oral, bloodborne, and zoonotic transmission. To date, there have been no published reports indicating SARS-CoV-2 transmission through feces or urine [85]. However, SARS-CoV-2 has been found in the feces of COVID-19 patients [60,95,96], leading to successful cultures of SARS-CoV-2 from stool specimens [97,98]. Additionally, levels of SARS-CoV-2 RNA concentrations in municipal wastewater parallel trends in local COVID-19 outbreaks, supporting an additional methodology for tracking SARS-CoV-2 levels in local human populations [99,100].

Previous studies have detected low concentrations of SARS-CoV-2 in plasma or serum [101]. The potential for bloodborne transmission remains unclear but it is unlikely given the low concentration of viral RNA detected from blood [102,103].

**Figure 1:**
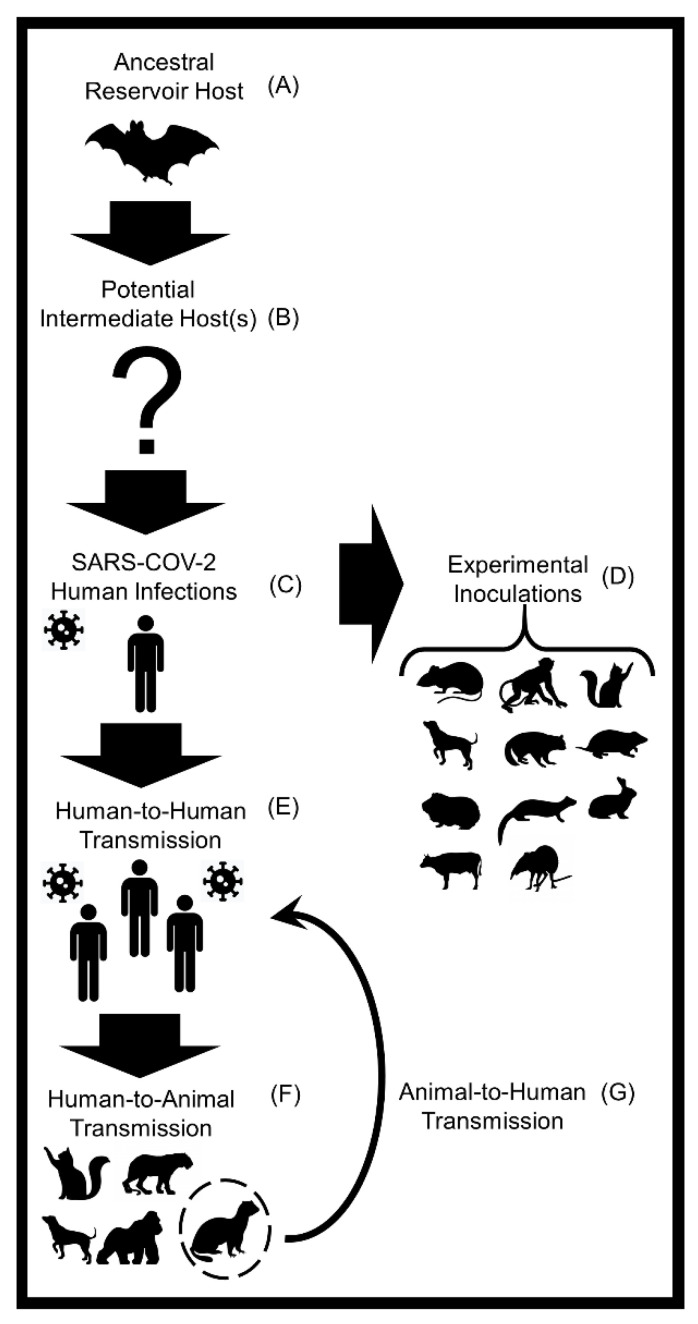
A conceptual diagram displaying the transmission of severe acute respiratory syndrome coronavirus 2 (SARS-CoV-2) among humans and various animal hosts. (**A**) Horseshoe bats (*Rhinolophus affinis*) are the most likely animal reservoir and ancestral hosts of the SARS-like CoV that gave rise to SARS-CoV-2 [53,105]. (**B**) A multitude of animals including mammals, birds, and reptiles have been proposed as potential intermediate hosts [38,39,41]. (**C**) SARS-CoV-2 was first reported in humans in December 2019 in Wuhan, China [106]. (**D**) Successful laboratory infections of SARS-CoV-2 have been reported in the following mammals: domestic dogs, domestic cats, ferrets, rabbits, raccoon dogs, hamsters, mice, tree shrews, cattle, and several species of non-human primates [107,108]. (**E**) In January 2020, the World Health Organization (WHO) first reported that human-to-human transmission of SARS-CoV-2 is feasible [109,110]. (**F**) Natural infections of SARS-CoV-2 in animals transmitted from humans (i.e., reverse zoonosis or anthroponosis) have been detected in domestic dogs and cats, domestic mink, ferrets, mice, hamsters, captive gorillas, and captive large cats (e.g., tigers and lions) [111,112]. (**G**) Evidence of SARS-CoV-2 spillback from domestic minks to humans and intraspecies transmission of SARS-CoV-2 among minks has been detected [46,47]. At this time these are the described transmission pathways and animals.

The most recent novel SARS-CoV-2 transmission pathway was described at the human-animal intersection, as current findings suggest a spillback and spillover potential of SARS-CoV-2, especially between humans and domestic mink [46,47] and between humans and companion cats [104]. This synthesis review serves to further evaluate the animal host diversity and zoonotic transmission potential of SARS-CoV-2 (Figure 1).

## 4. SARS-CoV-2 Spike Protein and ACE-2

**Figure 2:**
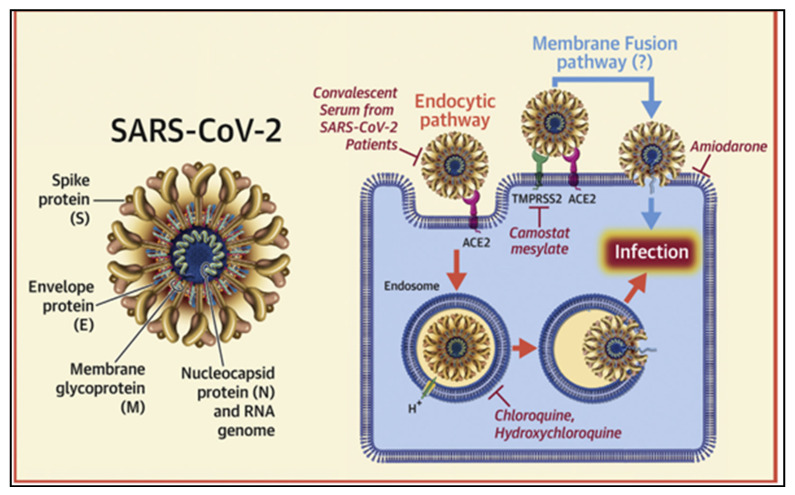
Structure of the severe acute respiratory syndrome coronavirus-2 (SARS-CoV-2). The virus is built up of four major structural proteins: the spike (S) protein; the nucleocapsid (N) protein; the membrane (M) protein; and the envelope (E) protein. The S protein is responsible for facilitating the entry of the CoV into the target cell. The routes employed by SARS-CoV include endocytosis and membrane fusion. The route employed by SARS-CoV-2 is via endocytosis; whether SARS-CoV-2 enters cells by membrane fusion is not known. Binding of the S protein of SARS-CoV to angiotensin-converting enzyme 2 (ACE-2) leads to the uptake of the virions into endosomes, where the viral S protein is activated by the pH-dependent cysteine protease cathepsin L. Activation of the S protein by cathepsin L can be blocked by bafilomycin A1 and ammonium chloride, which indirectly inhibit the activity of cathepsin L by interfering with endosomal acidification. Chloroquine and hydroxychloroquine are weak bases that diffuse into acidic cytoplasmic vesicles such as endosomes, lysosomes, or Golgi vesicles and thereby increase their pH. MDL28170 inhibits calpain and cathepsin L. SARS-CoV can also directly fuse with host cell membranes, after processing of the virus spike protein by transmembrane protease serine 2 (TMPRSS2), a type II cell membrane serine protease. Camostat mesylate is an orally active serine protease inhibitor. Figure and caption sourced from Ky and colleagues [114].

Across human and animal hosts, SARS-CoV-2 shares a common pathway of first docking to host cells via the spike protein (S-protein) [113] (Figure 2).

To enter host cells, the receptor-binding domain (RBD) of SARS-CoV-2 S-protein binds to the angiotensin-converting enzyme 2 (ACE-2) and then is processed by a cellular protease (TMPRSS2) [113,115] (Figure 2). TMPRSS2 facilitates the cleavage of the S-protein into subunit sites (S1/S2) allowing the fusion of viral and cellular membrane [116,117]. During the spillback of viral transmission from mink to humans, S-protein gene mutations were detected among infected humans [118]. Although this evidence is concerning, it presents little risk to the overall human population [119]. However, it highlights the necessity for virologists and epidemiologists to closely coordinate the exchange of scientific research. Theoretical approaches can be applied to predict the compatibility of S from SARS-CoV-2 to predict its binding to ACE-2 from other animal hosts.

Interactions between the S-protein and angiotensin I converting enzyme 2 (ACE-2) complexes have been modeled to predict potential vertebrate hosts susceptible to SARS-CoV-2 [120,121,122]. The sequence of ACE-2 is highly conserved throughout vertebrates and any species with cells that contain the ACE-2 receptors could potentially be susceptible [122,123]. Using a dataset of ACE-2 sequences, scientists have found a variety of animals that could potentially be susceptible to SARS-CoV-2 [122,124]. Critical amino acids that affect the binding of the S-protein to ACE-2 (e.g., K31, M82, N90, and K353) are commonly selected for analysis [122]. The amino acids were determined by identifying human binding residues of SARS-CoV-2 [54,122]. Furthermore, N-glycosylation motifs (e.g., N53) are conserved within all species and could potentially attach to the S-protein [54]. Based on protein modeling approaches, extensive vertebrate diversity has been reported as potentially susceptible to SARS-CoV-2 [122,125]. However, further experimental data will need to be conducted to confirm the results of the ACE-2 sequencing data [122].

## 5. Animal Host Diversity of SARS-CoV-2

Given the suspected animal origin of SARS-CoV-2, knowledge of susceptible animal species, intermediate hosts, reservoirs, and potential transmission routes between humans and various animal species is informative to both animal and public health authorities. While CoVs are generally host-specific, they can be transmitted to other species and adapted by frequent recombinant events [126,127] (Table 1).

The SARS-CoV and MERS-CoV infections provide examples of dynamic evolutionary histories and complex ecological transmission pathways [12,38,141].

Natural SARS-CoV-2 infections have been reported in domestic dogs, felids, and mustelids in China, Hong Kong, Europe, and the United States [40,142]. It is considered that a reverse zoonotic transmission route has been the driver in these field reports of naturally infected animals [46,47]. Except for farmed mink, it remains unclear if human-to-animal transmission could proceed to secondary infections from animals to humans [46] (Figure 1).

Experimental inoculation studies have reported infectivity of domestic dogs, domestic cats, ferrets, rabbits, hamsters, mice, and several species of non-human primates [107,108]. Host immune response and viral replication vary drastically between tested species [41]. SARS-CoV-2 ELISA kits have also been used as an approach to qualitatively assess the animal antibody detection potential [143]. Additionally, evaluation of ACE-2 receptors has revealed that amino acid residues responsible for viral binding are moderately conserved between humans and select animal groups [122]. Furthermore, vaccine development has been contingent on the use of rodents, felids, mustelids, and non-human primates as model organisms expressing ACE-2 receptors [107].

Overall, vulnerable susceptible animal groups include: (1) animals in contact with humans infected with SARS-CoV-2, (2) threatened or endangered species housed in rehabilitation or zoological centers, (3) temporary social or care settings where there is frequent contact between animals and humans, and (4) livestock housed in high densities on farms. Theoretical approaches, such as molecular tools [122] and protein modeling [34], provide alternative methodologies for predicting susceptible species. In particular, key residues of the ACE-2 receptor for recognizing S protein can be studied to predict potential animal hosts of SARS-CoV-2 [144,145,146]. Ultimately, an integrative framework that applies field, laboratory, and theoretical approaches provide a comprehensive framework for assessing potential hosts of SARS-CoV-2. We provide an overview of potential animal species that could potentially function as an evolutionary reservoir, intermediate, or susceptible host.

### 5.1. Bats

Bats have been identified as reservoirs for a variety of viral pathogens including Nipah virus, Hendra virus, influenza, Ebola, rabies, and CoVs [147]. It is hypothesized that a virus-tolerant phenotype in bats has facilitated the co-evolution of bat hosts with viral pathogens [148]. Molecular epidemiological studies of the origins of CoVs have linked SARS-CoV-like CoVs in bats as the reservoirs to SARS-CoV, MERS-CoV, and endemic HCoV-NL63 and HCoV-229E [16,149]. Recent research suggests that bats are the evolutionary reservoir to SARS-CoV-2 [105,141,150]. Recent studies suggest that a SARS-CoV-like CoV collected from horseshoe bats (*Rhinolophus affinis*) (RaTG13) is approximately 96% similar to SARS-CoV-2 [105,141,151]. Moreover, an insertion between the cleavage site S1 and S2 in the SAR-CoV-2 genome has also been found in another horseshoe bat species (*R. malaynus*), indicating that at least two bat species were likely ancestral reservoirs of SARS-CoV-2 (CoV-RaTG13). Phylogenetic reconstruction of the SARS-CoV-2 lineage reveals that the most recent divergence from SARS-CoV-like CoVs occurred at least several decades ago, providing evidence that the ancestral lineage of SARS-CoV-2 has been circulating in bat populations for decades [27]. To date, there is no definitive evidence that bats can directly transmit SARS-CoV-2 to humans [152]. Increased and continuous surveillance of free-ranging bats is critically needed globally. Olival and colleagues [153] predicted that over 40 species of bats living in temperate zones within North America could be susceptible to SARS-CoV-2, and could facilitate a potential viral spill-back into novel wildlife reservoirs [153].

Despite the significance of bats in SARS-CoV-2 epidemiology, several key observations suggest that an unknown animal might serve as an intermediate host for SARS-CoV-2 transmission between bat and human populations [150] (Figure 1). First, the COVID-19 outbreak occurred in December 2019 when most local bat species of Wuhan remain in hibernation [150]. Second, no bats were sold or held captive at the Huanan seafood market, while many non-aquatic wildlife species were available for purchase [150]. Third, the viral sequence samples from SARS-CoV-2 and the most closely related sequences are nearly 96% similar [150]. These molecular results could demonstrate that bats are not direct ancestors of SARS-CoV-2 [150,154]. Collectively, these findings underline the necessity for evaluating potential intermediate animal hosts of SARS-CoV-2.

### 5.2. Pangolins

Previous research studies point to Malayan pangolins (*Manis javanica*) as a potential intermediate host of SARS-CoV-2 [32,37]. On 24 October 2019, the first reported SARS-CoV-like CoV, named Pangolin-CoV, was detected in two dead Malayan pangolins from the Guandong Wildlife Rescue Center of China [155]. The viral genomic material collected from the site was approximately 80 to 91% similar to known CoVs and was speculated to be closely related to novel SARS-CoV-2 [156]. Shortly after RaTG13, Pangolin-CoV is the second closest related CoV to SARS-CoV-2 that was reported [156]. Additionally, the S1 protein of Pangolin-CoV is more closely related to SARS-CoV-2 than RaTG13 [156,157,158]. Amino acid sequences involved with the interaction of ACE-2 with humans are comparable to those of Pangolin-CoV and SARS-CoV-2 [32,151]. Despite these findings, subsequent phylogenetic analysis reveals that the margin of genetic variation between pangolin-CoV-2020 and SARS-CoV is too large to be the direct descendant of SARS-CoV-2 [155]. It is contested whether pangolins are natural hosts for *Betacoronaviurses* [120,159] or simply dead-end hosts [35]. Further surveillance of CoVs in pangolins could improve knowledge in the evolutionary history of SARS-CoV-2 [160,161] because pangolins might serve as a link that may have facilitated the initial SARS-CoV-2 infections in human populations [120,162].

### 5.3. Felids

Felids are susceptible to a variety of CoVs including SARS-CoV-like CoVs (*Betacoronaviruses*) and feline coronaviruses (*Alphacoronaviruses*). Feline CoVs have been documented to infect only domestic and wild felids, and there is limited evidence demonstrating that previous exposure to feline coronaviruses is protective against SARS-CoV-2 infections [163]. Currently, several studies documented felids testing positive with SARS-CoV-2 throughout the world [86]. In most of these cases, the cats were either domestic or captive and had recent contact with known SARS-CoV-2 human cases [53,86,164,165]. A recent, longitudinal surveillance study conducted in Texas reported that over 25% of households with at least one SARS-Cov-2-infected human had one infected companion dog or cat [104]. Several observational studies have reported pet cats infected with SARS-CoV-2, likely due to reverse zoonotic transmission from infected owners. A cohort study in Wuhan, China detected SARS-CoV-2 seropositivity in abandoned cats in shelters, in cats from households of SARS-CoV-2 patients, and cats taken to veterinary clinics [166]. In Belgium, a cat was tested positive for SARS-CoV-2 that belonged to an owner who tested positive for SAR-CoV-2 after traveling from northern Italy [167]. The cat also exhibited respiratory symptoms, nausea, and diarrhea [167]. In another study, laboratory results revealed that the vomit and feces of a cat exhibited a high concentration of SARS-CoV-2 RNA [168]. In Hong Kong, the Agriculture, Fisheries and Conservation Department (AFCD) reported on 31 March 2020, that SARS-CoV-2 had been detected in the oral cavity, nasal, and rectal samples of a cat [169]. The owner of the infected cat had been previously hospitalized for SARS-CoV-2 infection with no clinical signs. Additionally, a previous experimental study described that cats that recovered from SARS-CoV-2 and were reinoculated with SARS-CoV-2 had a strong protective immune response to prevent them from reinfection [170]. In another study from Spain, an asymptomatic cat tested positive for SARS-CoV-2 and was euthanized because of cardiomyopathy [171].

Susceptibility to various CoVs has led to the selection of domestic cats as animal models in CoV research [107,166]. Experimental studies have documented cats are highly suspectable to SARS-CoV-2 and able to transmit the virus to naïve conspecifics [53,170,172]. In a laboratory pilot study, domestic cats shed the virus for up to 5 days and infected naïve cats with the virus through direct contact [170]. Results from Gaudreault and team members [173] corroborated these findings by showing that inoculated cats were able to infect other cats and remained asymptomatic throughout the study. Inoculated cats exhibit asymptomatic to moderate COVID-19 symptoms [53,172]. Despite increased understanding from experimental research on felids, cats are not standard experimental animals and are difficult to handle in biosafety 3 settings [174]. Additional field investigations could circumvent these logistical challenges by monitoring environmental contamination (e.g., litter box, food, water bowls) or transmission efficiency between owners and domestic cats [174,175].

Felids housed in zoos are at risk from potential SARS-CoV-2 exposure from infected caretakers. On 27 March 2020, the United States Department of Agriculture (USDA) reported the first animal in the United States and first non-domestic species in the world, a Malayan tiger (*Panthera tigris*) in the Bronx Zoo, New York, to test positive for SARS-CoV-2 was [86,112]. On 3 April 2020, an additional Malayan tiger, two Amur tigers (*Panthera tigris altaica*) housed in the same building but different enclosures, and three African lions (*Panthera leo krugeri*) developed similar respiratory symptoms [86,112]. The presence of viral RNA in feces was consistent in all of the clinical cases and persisted for up to 35 days after cessation of respiratory symptoms in 1 Amur tiger [176]. Follow-up analysis led to the identification of 9 whole SARS-CoV-2 genomes from tigers, lions, and their keepers [112]. Sequencing displayed two distinct genotypes between the lions and tigers, suggesting that human-to-tiger transmission occurred in two separate events [113]. The exact epidemiological driver(s) and evidence that facilitated the human-to-tiger transmission such as direct (e.g., animal handling), indirect (e.g., food preparation/handling, fomite), or subsequent tiger-to-tiger transmission (e.g., aerosol, respiratory droplet) remains unclear [112].

### 5.4. Mustelids

In 2006, ferret enteric CoVs (FRECV) RNA was detected in domestic ferrets (*Mustela putorius furo*) [177]. As a member of *Alphacoronavirus*, FRECV is evolutionarily distant from SARS-CoV-2. However, contextualization of ferret susceptibility to CoVs is important for studying SARS-CoV-2. Ferrets have been proposed as potential natural hosts for SARS-CoV-2 because many laboratory inoculation experiments with SARS-CoV-2 were reported [178,179,180,181,182]. In laboratory settings, SARS-CoV-2 infects the upper respiratory tract of ferrets but it does not effectively spread between individuals [53]. Laboratory infected ferrets shed SARS-CoV-2 via saliva, urine, and nasal washes and potential airborne transmission was possible to naïve ferrets [180,183]. The advantages of using ferrets as a model animal for vaccine development are that they can be used for evaluating cough and fever symptoms and they have an extensive history of respiratory viral research [184,185]. The downsides of ferrets as a model animal are that it remains unclear if edema and serious lung infection can be caused by SARS-CoV-2 in them [107]. To date, there has not been a field study that documented natural infection of SARS-CoV-2 in ferrets. In one household with 29 ferrets and 2 infected humans, there was no evidence of human-to-ferret transmission based on RT-PCR and ELISA tests [186].

In contrast to ferrets, minks are susceptible to natural infections and can spread SARS-CoV-2 to other minks, other animal species, and humans [46,187]. In mid-April 2020, SARS-CoV-2 outbreaks were reported in two mink farms in the Province North Brabant, Netherlands [187]. The two farms were 14 km apart with no exchange of workers, vehicles, or animals between them [187]. Among the infected minks, the cause of death was mostly due to interstitial pneumonia but also a few individuals exhibited sepsis, Aleutian disease, lung edema with congestion, and dystocia [187]. Viral RNA was present in the conchae, lung, throat swab, and rectal swab of all infected minks sampled, and at least one farm worker was diagnosed with SARS-CoV-2 before the outbreaks [187]. Inhalable dust in the farmhouses contained viral RNA, suggesting a potential exposure source for workers [187]. Follow-up whole genome sequencing analysis confirmed that SARS-CoV-2 was likely first introduced by humans and thereafter evolved due to widespread circulation among minks for several weeks before the onset of outbreaks [46]. Additionally, 68% of the tested mink farm residents and workers have shown SARS-CoV-2 infections [46]. Genomic analysis of this population displayed a unique animal sequence signature, suggesting a mink-to-human spillover within mink farms [46]. In addition to genomic and epidemiological evidence, an observational study noted that mink likely transmitted SARS-CoV2 to surrounding feral cats [47]. Tens of thousands of minks were euthanized from these two farms to limit further disease spread [47]. To prevent further SARS-CoV-2 transmission and spread among humans and ferrets, and prevent the emergence of novel viral strains, the Dutch government issued a mandate to close all mink farming operations by March 2021 [188]. The spillover of SARS-CoV-2 to mink from humans and then the spillback from mink to humans is not a novel transmission pathway and since documented in the Netherlands, it has also been found in Denmark, Italy, Greece, Spain, Sweden, and the United States [189,190,191].

### 5.5. Rodents

Previous studies suggest that endemic HCoV-OC43 and HCoV-HKU1 originated from primordial associations of both viruses that first existed in rodents [6]. Wildlife field sampling of various rodent species led to the discovery of the novel Luncheng Rn rat coronavirus (LRNV) [131]. Multiple viral recombinant events among several rodent species could suggest that there is an unrecognized viral diversity in rodents [131,192].

Similar to North American bats, free-ranging rodents could also be at risk of reverse zoonotic transmission of SARS-CoV-2 [193]. There is a concern that if spillback of SARS-CoV-2 were to infect a naïve wild rodent and establish its circulation in natural populations, then these populations could potentially maintain the virus and transmit it to human populations [38]. North American deer mice (*Peromyscus maniculatus*) and closely related rodents of the Cricetidae family carry 18 of the 20 critical residues within the ACE-2 receptor, facilitating SARS-CoV-2 spike protein binding [194]. As the most studied and abundant mammal species in North America, [195], deer mice are known reservoirs for other zoonotic pathogens including *Borrelia burgodferi* (Lyme disease), *Yersinia pestis* (plague), and Sin Nombre orthohantavirus (hantavirus pulmonary syndrome) [196,197] that could spill over into human populations. Experimental inoculations demonstrated that deer mice are susceptible to SARS-CoV-2 without showing clinical signs of infection, and can spread SARS-CoV-2 to uninoculated susceptible mice [194]. However, other rodent species displayed negative SARS-CoV-2 ELISA test results [143]. Further research is necessary to evaluate the potential spread of SARS-CoV-2 to deer mice, and from deer mice to house mice and subsequently to humans.

Rodents have also been used as model organisms in SARS-CoV-2 research to better understand infectivity, virulence, pathogenicity, and host-pathogen interactions because they are not only susceptible to SARS-CoV-2 but also exhibit key features of the human disease [41,107,108]. For example, mouse fusion proteins instill evidence for domestic animal susceptibilities such as camels, cattle, horses, goats, sheep, pigs, cats, and rabbits and support efficient transmission of SARS-CoV-2, SARS-CoV, and Bat-nCoV RaTG13 [198]. Furthermore, rodents have been used as model organisms for SARS-CoV-2 vaccine development [199,200,201,202].

Currently, infection of hamsters by SARS-CoV-2 has only occurred during experimental infections [203]. Hamsters have been used as useful model animals for studying pathology and host-pathogen interactions of many coronaviruses [41,107]. When compared to mouse models, hamsters exhibit greater viral shed rates, higher viral concentration, and longer infectivity durations [204]. Syrian hamsters (*Mesocricetus auratus*) have been recognized as one of the key model animals for SARS-CoV-2 research [20,41,107]. When inoculated with SARS-CoV-2, hamsters develop severe lung lesions that are similar to humans hospitalized with SARS-CoV-2 infections [205]. Imai and colleagues [205] conducted an experimental trial and described the following key findings: (1) inoculated hamsters showed pathological signs of SARS-CoV-2 infection, (2) infected hamsters mounted an antibody response to SARS-CoV-2, (3) produced antibodies were protective against reinfection, and (4) antibodies transferred to naïve hamsters were effective in preventing SARS-CoV-2 infections. Another study conducted by Sia and colleagues [206] reported that inoculated hamsters infected co-housed naïve hamsters. Although inoculated and co-housed hamsters lost >10% body mass, all studied hamsters recovered after the infections [206].

### 5.6. Eulipotyphlans

Only a few studies have analyzed CoVs in eulipotyphlans, in particular in hedgehogs and shrews [6]. Although there are fewer species in Eulipotyphla compared to Rodentia (rodents) and Chioptera (bats), various CoVs have been linked to several CoVs clades including some that have public health importance such as SARS-CoV [207,208,209]. Eulipotyphlans are also known reservoirs for a variety of pathogens such as BoDV-1 (Borna virus), *Bartonella* spp., rotaviruses, and hantavirus [210,211,212,213,214]. It is predicted that CoVs of hedgehogs might recombine with bat CoVs, facilitating the emergence of novel CoVs that could infect new hosts [215]. Although SARS-CoV-2 has not been reported in eulipotyphlans, phylogenetic analysis of ACE-2 protein sequences of Amur hedgehog (*Erinaceous amurensis*) displays close relatedness to that of the Chinese rufous horseshoe bat (*R. sinicus*) [216].

### 5.7. Tree Shrews

Belonging to the order Scandentia, Chinese tree shrews (*Tupaia belangeri chinensis*) are evolutionarily closely related to primates [217]. These squirrel-sized mammals are distributed throughout southeast Asia and southwest China [218]. Tree shrews have been used as model animals for studying viral infections such as influenza [219,220], HSV-1 [221], hepatitis B and C virus [222,223], and hand-foot-mouth disease [156,224]. Phylogenetic sequencing of their ACE-2 receptors reveals a high sequence similarity between tree shrews and humans (up to 81%) [105]. These observations have been supported by laboratory inoculations that have reported tree shrews can not only be infected by SARS-CoV-2 but also can develop minor signs of respiratory infections [225,226]. Despite displaying subtle pathology, it is speculated that tree shrews might serve as an intermediate host or as an asymptomatic carrier of SARS-CoV-2 [227].

### 5.8. Lagomorphs

Members of the order of Lagomorpha, such as pikas and rabbits, are known reservoirs for a variety of viral pathogens such as influenza, astrovirus, rotavirus A, narmovirus, hepatitis E virus [208,228,229]. Free-ranging European rabbits (*Oryctolagus cuniculus*) are of special interest as reservoirs for CoVs because of the recent detection of betacoronaviruses in domestic rabbits in China [33]. Theoretical protein models provide the first reported potential of SARS-CoV-2 binding to the ACE-2 receptors of lagomorphs [122,230,231,232]. Preliminary laboratory work by Mykytyn and colleagues [233] reported an experimental infection and viral shedding of New Zealand white rabbits (*Oryctolagus cuniculus*) [233]. Viral infectivity is considered to be lower in rabbits compared to hamsters and ferrets [233]. Similar to mink [47], there is a concern that farmed rabbits could facilitate the transmission and spread of SARS-CoV-2 to human populations [234]. Further surveillance and experimental research of lagomorphs are needed to understand transmission dynamics among humans and rabbits to prevent potential outbreaks within both wildlife and captively raised lagomorphs and humans.

### 5.9. Canids

Preliminary case reports have documented SARS-CoV-2 transmission among domestic dogs and their owners that tested positive for SARS-CoV-2. On 28 February 2020, in Hong Kong, the first domestic dog that tested positive for SARS-CoV-2 was reported in a Pomeranian dog belonging to an owner that was previously tested positive for SARS-CoV-2 [235]. Subsequent asymptomatic dog infections in Hong Kong were reported; all sharing living environment with at least one SARS-CoV-2-positive owner [175,236,237]. During the same period, in the Netherlands, one pet dog was reported positive for SARS-CoV-2 and its owner had previously been hospitalized for SARS-CoV-2 [238]. It was demonstrated that SARS-CoV-2 can harm a dog’s smell (i.e., hyposmia, anosmia) [45]. A laboratory study in five-week-old beagles provided evidence for seroconversion in two dogs and viral RNA was detected in rectal swabs two days post-inoculation [53]. These findings, given that beagles are service dogs, underscores the potential security risk as service dogs are commonly deployed throughout the world to assist with patrol, tracking, and scent detection [239,240]. Field epidemiological investigations provided evidence that SARS-CoV-2 infection in domestic dogs occurred from their infected owners [235].

During the 2002–2004 SARS-CoV pandemic, researchers isolated SARS-CoV in raccoon dogs (*Nyctereutes procyonoides*) in China, providing evidence that these canids might have functioned as an intermediate host for the CoV [241]. Experimentally, raccoon dogs have been documented to succumb to infection and to transmit SARS-CoV-2 to naïve raccoon dogs [242]. Within this first reported study, none of the infected raccoon dogs exhibited symptoms and virus tissue lesions occurred in the nasal conchae [242]. Moreover, the ACE-2 of raccoon dogs is identical to the ACE-2 of domestic dogs [243]. Producers in China house over 14 million raccoon dogs, equating to nearly 99% of the global raccoon dog fur trade market [244].

Although dogs ACE-2 proteins can function as receptors to SARS-CoV-2, experimental inoculations demonstrate a low susceptibility of dogs to SARS-CoV-2 [53]. Moreover, in dogs, SARS-CoV-2 tends to congregate in different organs (i.e., kidney and heart) than in humans (i.e., lungs) [243]. Co-expression of key receptors, ACE-2 and TMPRSS2, is rarely detected in dog lungs [245]. Furthermore, molecular evolutionary analysis of ACE-2 receptors revealed that the key amino acids are present in felids and humans but not in canids [246]. Consequently, these results combined with the description of the natural infections suggest that canids are likely not a reservoir for SARS-CoV-2 but rather act as an intermediate or dead-end host with limited potential for viral shedding or transmission.

### 5.10. Non-Human Primates

Non-human primates have been used to study infectious diseases in humans because of their close evolutionary relatedness [247]. Non-human primates have been linked to the transmission of many infectious diseases in human populations such as human immunodeficiency virus (HIV), herpes B virus, monkeypox, yellow fever, and Ebola [248,249]. To date, SARS-CoV-2 experimental studies have been reported in rhesus macaques (*Macaca mulatta*) [250,251,252], cynomolgus macaques (*Macaca fascicularis*) [253], and green monkeys (*Chlorocebus sabaeus*) [254]. Regarding experimental studies with SARS-CoV, green monkeys, common marmosets (*Callithrix jacchus*), squirrel monkeys (*Saimiri* spp.), and moustached tamarins (*Saguinus mystax*) have been used [255]. Experimental comparisons by Lu and colleagues [256] demonstrated that rhesus macaques exhibited higher upper respiratory viral shedding than cynomolgus macaques and common marmosets. An anti-spike antibody (LY-COV555) collected from a human previously infected with SARS-CoV-2 protects the upper and lower respiratory tracts from infection of SARS-CoV-2 in rhesus monkeys [257]. Further study of rhesus macaques has been used to show protective coverage against SARS-CoV-2 with a single dose of adenovirus serotype 26 (Ad26) vector-based vaccine [258]. In the United States, several zoos have documented SARS-CoV-2 infections among captive gorillas [259,260]. Although gorillas housed in zoos are at a lower risk of severe morbidity and mortality because of their access to veterinary care, wild gorilla populations are more vulnerable since human respiratory diseases are the leading causes of mortality among them [261]. As documented [107,262,263], it is evident that non-human primates play a crucial role as model animals for SARS-CoV-2 research and vaccine development.

### 5.11. Livestock

It is noteworthy to evaluate livestock species as hosts for SARS-CoV-2 because of their close and frequent contact with humans. Previous SARS-CoV-2 ELISA antibody tests revealed that pigs, cows, sheep, horses, and alpacas display a negative antibody response [143]. The potential spread of SARS-CoV-2 into livestock poses food security, economic, and public health risks. Further investigation of SARS-CoV-2 within livestock could limit the potential spread and delineate the best diagnostic tools for surveillance [45].

Dromedary camels (*Camelus dromedarius*) are of special importance because they are the natural reservoir of MERS-CoV [264]. Transmissions including camelid-to-human [265,266] and camelid-to-non-camelid domestic animals have been documented [267]. Dromedary camels have been considered model animals for vaccine development because they shed large MERS-CoV concentrations in their upper respiratory tract and show mild symptoms [268]. Additionally, 229E-like [136] and HCoV-HKU-like CoVs [269] are found in camels, but it remains unclear the direction of the cross-species transmission [136]. Camel susceptibility to SARS-CoV-2 is unlikely [152]. However, preliminary work by Gai and colleagues [270] applied antibodies collected from camels immunized with SARS-CoV-2 spike receptor-binding domain (RBD) to block the interaction with human ACE-2 receptors.

Alpacas (*Vicugna pacos*) are also susceptible to natural MERS-CoV infections [271,272]. Single domain antibody fragment, Ty1, from alpaca targets RBD of SARS-CoV-2 spike proteins, and thus preventing ACE-2 engagement [273,274]. Additionally, 229E-related alpaca virus occupies an intermediate phylogenetic position between bats and humans, suggesting previous viral recombination events [132].

Bovine coronaviruses are widely distributed throughout the world, causing respiratory infections in cattle (*Bos taurus*) [275,276]. Sequencing of ACE-2 receptors in domestic cows and buffalos reveals potential use by SARS-CoV-2 [277]. Ancestral human CoV-OC43-like CoVs were previously detected in cattle and swine [268]. Ulrich and colleagues [278] inoculated two cattle that comingled with three uninoculated cattle. Interestingly, the two inoculated cattle exhibited viral replication even though both were previously infected by a bovine betacoronavirus [278]. The uninoculated cattle did not become infected [278]. These findings corroborate with theoretical modeling predicting a medium susceptibility of cattle based on the ACE-2 cellular receptor for SARS-CoV-2 [122].

Multiple experimental inoculations revealed that domestic pigs (*Sus scrofa domesticus*) exhibit minimal susceptibility to SARS-CoV-2 [53,279,280]. Additional refuting evidence reports higher mRNA levels in organs such as kidneys and heart and almost no mRNA in the expected site of infection, the respiratory tract [243]. Similarly, pigs were not considered natural hosts of MERS-CoV-2 because infection of the virus did not cause disease and causes low viral shedding rates [281]. Pigs are evolutionarily important to study because of their genetic relatedness to humans relative to other livestock species [282,283]. Among CoVs, pig- and human-specific CoVs appear to have distinct host ranges with minimal overlap [284]. However, pigs have a variety of cell types expressing SARS-ACE-2 [245], supporting an efficient entry of SARS-CoV-2, SARS-CoV, and Bat-nCoV RaTG13 [198]. Moreover, the ACE-2 receptors of pigs could potentially be used by SARS-CoV-2 [277]. It is speculated that pig ACE-2 receptors could genetically recombine to mediate SARS-CoV-2 entry into other species similar to other swine recombinant events [125].

### 5.12. Aves

Domestic and wild birds are susceptible to a variety of CoVs within the *Gammavirus* and *Deltavirus* genera [3]. Interspecies transmission of CoVs has been reported between wild birds and domestic poultry [285]. In contrast, no avian species have been described to be susceptible to SARS-CoV-2. Poultry, including chickens, turkeys, geese, ducks, quail, and pigeons do not exhibit any immunological response or viral replication when challenged with SARS-CoV-2 [53,245,286,287]. Despite these findings, a phylogenetic analysis conducted by Qui and colleagues [277] identified that pigeon ACE-2 receptors might be utilized by SARS-CoV-2.

### 5.13. Reptilia

Although no CoV has ever been reported to infect reptiles [160], Testudines and Serpentes have been proposed as potential intermediate hosts for SARS-CoV-2 due to the interaction between key amino acids of S protein RBD and ACE-2 [288]. In terms of testudines, the western painted turtle (*Chrysemys picta bellii*), the green sea turtle (*Chelonia mydas*), and the Chinese softshell turtle (*Pelodiscus sinensis*) share unique amino acids within RBD domain (ASn501) and ACE-2 receptor (sites 41 and 353) more closely related to pangolins and humans than bats [288]. However, these speculations have been refuted by many because analysis of the S protein with key residues in ACE-2 from Testudines was abolished [289].

Serpentes, the many-branded krait (*Bungarus multicinctus*) and the Chinese cobra (*Naja atra*), have been proposed as wildlife reservoirs of SARS-CoV-2 because of similar virus codon usage patterns to that of humans [290]. These predictions have received great scrutiny for limitations in study design due to a lack of reproducibility in results because of various factorings including limited protein sequences, small vertebrate diversity analysis, and outdated codon usage database [144,255,291].

## 6. Conclusions

The COVID-19 pandemic highlights the societal, economic, and public health impacts of animal origin virus spillover events. Although SARS-CoV-2 is primarily spread via a human-to-human transmission [292,293], there is growing evidence for human-to-animal [38,142,294], animal-to-animal [53,112], and in select case studies of spillback via an animal-to-human transmission [46,187]. Here, we reviewed the infectivity and transmission potential of SARS-CoV-2 natural cases identified in lions, tigers, domestic cats, domestic dogs, farmed mink, and deer mice. In experimental settings, SARS-CoV-2 has been reported to successfully infect raccoon dogs, Syrian hamsters, fruit bats, tree shrews, New Zealand white rabbits, ferrets, cattle, and non-human primates. Theoretical approaches have hypothesized the potential for other livestock, avian, and reptile hosts. Domestic animals and wildlife housed in zoological facilities are at a higher risk of exposure from frequent contact with SARS-CoV-2-infected humans [39].

Monitoring the epidemiological dynamics and disease ecology of humans and animals could enhance overall public health interventions while also preventing pathogen establishment in novel animal hosts [153,295]. Mustelids, specifically domestic mink, present the greatest public health risk because of molecular and epidemiological evidence of SARS-CoV-2 transmission from mink to other mink, to cats, and humans [46,47].

The Netherlands represents a model for other national agricultural programs by banning mink farming to prevent the establishment of SARS-CoV-2 in mink [188,296]. Establishment in animal reservoirs could lead to the evolution and spread of novel SARS-CoV-2 sequences that could lessen the efficacy of human vaccines. Lessons from MERS-CoV have shown how a CoV can remain endemic in human populations due to continued resurgence via spillover from dromedary camels to humans [297,298,299]. Public health implementation programs also carry value to conservation biology as all members of Felidae, many of which are endangered, are susceptible to SARS-CoV-2 [112,246,300].

Several public health programs have initiated active surveillance programs to assess not only humans with SARS-CoV-2 but also their companion animals [104,166,235]. Specifically, companion animals exhibit mild to no symptoms when challenged with SARS-CoV-2 [170,268]. Moreover, companion animals could serve as sentinel hosts for monitoring SARS-CoV-2 in the human population [164,301]. Similarly, previous surveillance systems have monitored cat shelters for low-pathogenic avian influenza (LPAI) A(H7N2) virus in cat shelters [302] along with West Nile virus and Usutu virus in chicken flocks [303,304]. These successfully applied implementation programs could serve as examples for comprehensive human and animal SARS-CoV-2 surveillance.

This pressing issue exemplifies the importance of instituting global SARS-CoV-2 surveillance involving physicians, veterinarians, ecologists, microbiologists, and epidemiologists to not only monitor but also implement public health interventions to prevent the establishment of SARS-CoV-2 in novel animal hosts and limit animal to human spread [54,153]. We urge the application of a “One Health” approach to foster cross-disciplinary scientific collaborations and to support coordinated effective preventive medicine implementations.

## Figures and Tables

**Table 1:** Examples of coronavirus recombinant events in wildlife, domestic animals, and humans.

Genus	Species	Recombinant Events	Hosts
*Alphacoronavirus*	SADS-CoV	HKU2-related bat COVs from horseshoe bats (*Rhinolophus sinicus, Rhinolopus pusillus, Rhinolopus rex, Rhinolopus affinus*) with swine acute diarrhea syndrome coronavirus (SADS-CoV) [128].	Domestic pigs
	S INDEL PEDV	Transmissible gastroenteritis virus mutant with recombination of G1a CV777-lineage classical and the G2 strain of porcine epidemic diarrhea virus (PEDV) [129].	Domestic pigs
	Rhinolophus bat coronavirus HKU2	Evidence of previous recombinant events with HKU2 from horseshoe bats shares 15-amino acid peptide corresponding within the RBM of the spike protein of SARS-CoV [130].	Horseshoe bats (*Rhinolophus sinicus*)
	Lucheng Rn rat coronavirus (LRNV)	Recombinant origin due to its N gene sequence more closely related to the genus *Betacoronavirus* than *Alphacoronavirus.* Also formed a divergent lineage in S gene tree with horseshoe bat coronavirus HKU2 [131].	Brown rats (*Rattus norvegicus*)
	HCoV-229E	Recombination event between alpaca HCoV-229E and bat 229E-related CoVs [132].	Noack’s round-leaf bats (*Hipposideros* cf. *ruber*) and Aba round leaf bats (*Hipposideros abae*)
	Canine Coronavirus type 1 (CCoV-II)	Recombination event involving CCoV-II and porcine transmissible gastroenteritis virus (TGEV) [133]	Domestic dogs
	Ferret Coronavirus (FRCoVs)	Comparison of FRCoV with ferret systemic coronavirus and ferret enteric coronavirus revealed that recombination occurred in the spike, 3c, and envelope genes occurred between different FRCoVs [134].	Domesticated ferrets (*Mustela putorius*)
*Betacoronavirus*	SARS-CoV-2	Horseshoe bats (*Rhinolophus* spp.) (RaTG13) and potentially Malayan pangolins (*Manis javanica*) (PCoV) [27].	Humans and cats (*Felidae*)
	SARS-CoV	SARS-CoV-like viruses from horseshoe bats (*Rhinolophus macrotis, Rousettus leschenaulti, Rhinolophus pearsoni, Rhinolophus pussilus*) and humans [135].	Horseshoe bats (*Rhinolophus* spp.), Himalayan palm civets (*Paguma larvata*), raccoon dogs (*Nyctereutes procyonoides)*
	MERS-CoV	Humans and dromedary camels (*Camelus dromedarius*) [136].	Bats *(Neoromicia capensis*, *Vespertilio superans*), dromedary camels (*Camelus dromedarius*), and European hedgehogs (*Erinaceus europaeus*)
	Bovine CoV (BCoV)	There is a putative recombinant detection only in the BCoV strain HEC 4408 with human CoV OC43 [137].	Waterbuck (*Kobus ellypsiprimus*), sambar deer (*Cervus unicolor*), white-tailed deer (*Odocoileus virginianus*), elk (*Cervus elephus*), giraffe (*Giraffa camelopardalis*), and sable antelopes (*Hipotragus niger*)
*Deltacoronavirus*	Wigeon coronavirus HKU20	NS7b of WiCoV HKU20 and CMCoV HKU21, and NS7d of WiCoV HKU20, were also found to be homologous to the NS3b of IBV and hypothetical protein of goose coronavirus, respectively [138].	Wigeons (*Mareca* spp.)
	Bulbul Coronavirus HKU11	NS7a of NHCoV HKU19 was found to be homologous to the NS7a of BuCoV HKU11, ThCoV HKU12, and MunCoV HKU13 [138].	Bulbuls (*Pycnonotidae*)
	Porcine coronavirus HKU15	PorCoV HKU15 contains a stem-loop II motif, a conserved RNA element downstream of N and upstream of the poly(A) tail, similar to those in Infectious Bronchitis Virus, TCoV, SARSr-Rh-BatCoV, and SARS-CoV [138].	Domestic pigs
*Gammacoronavirus*	Beluga whale CoV (BWCoV)	Beluga whale BWCoV SW1 (*Delphinapterus leucas*) and bottlenose dolphin (*Tursiops truncatus*) BDCoV HKU22 exhibit high similarity [139].	Beluga whales (*Delphinapterus leucas*)
	Infectious bronchitis virus (IBV)	Evidence of recombinant events can be seen from the high similarity in IBV strains of chicken and turkey CoVs [140].	Turkey (*Meleagris spp.*), Goose (*Anser* spp.), Duck (*Anas* spp.), and Pigeons (Columbidae)

## Data Availability

No new data were created or analyzed in this study. Data sharing is not applicable to this article.
